# Variability analysis in PM2.5 monitoring

**DOI:** 10.1016/j.dib.2019.103774

**Published:** 2019-03-14

**Authors:** Rashmi Bhardwaj, Dimple Pruthi

**Affiliations:** Guru Gobind Singh Indraprastha University, India

## Abstract

United States Environmental Protection Agency (US EPA) and Central Pollution Control Board (CPCB) are two major air quality monitoring agencies in India that measure the concentration of particulate matter of size up to 2.5 μm (PM2.5). PM2.5 study over southern Asia has significance from the environment and ecosystem viewpoint (Abdullah et al.,2007; Dockery and Stone, 2007). In order to raise alert and controlling of pollutants, not only forecasting but the accuracy of forecasting has attracted attentions from various departments of research and air quality monitoring agencies. Quest for reducing error in forecasting has never come to pause. The precursor in forecasting is data monitoring. Keeping in focus the initial phase of data analysis, PM2.5 concentration was collected from both agencies within an area of radius 3.1 miles for the year 2016. Using the data, variability analysis is carried out for the efficiency of vital environment protection agencies.

**Table:** Specifications table

Subject area	*Mathematics*
More specific subject area	*Statistics*
Type of data	*Table*
How data was acquired	*www.cpcb.nic.in**,**www.epa.gov*
Data format	*Raw, processed and analyzed.*
Experimental factors	*Investigation of monitoring variability and forecasting*
Experimental features	*Air quality Index assessment of PM2.5 monitored by US EPA and CPCB*
Data source location	*R. K. Puram, India*
Data accessibility	*www.cpcb.nic.in*, *www.epa.gov*
Related research article	*L. Zhang, J. Lin, R. Qiu, X. Hu, H. Zhang, Q. Chen, H. Tan, D. Lin, J. Wang, Trend analysis and forecast of PM2.5 in Fuzhou, China using the ARIMA model, Ecol. Indic. 95 (2018) 702–710.*

**Table:** 

**Value of the data** • The dataset used in this article reflects the variability in monitoring by United States Environmental Protection Agency and Central Pollution Control Board. • Air Quality Index calculated using data gives status of air we breathe in. • The dataset will help to determine the effect of fine particles. • The information contained in this article can be used to assess environment impact. • The information provided can form the basis for issuing health advisory.

## 1 Data

**Fig. 1:**
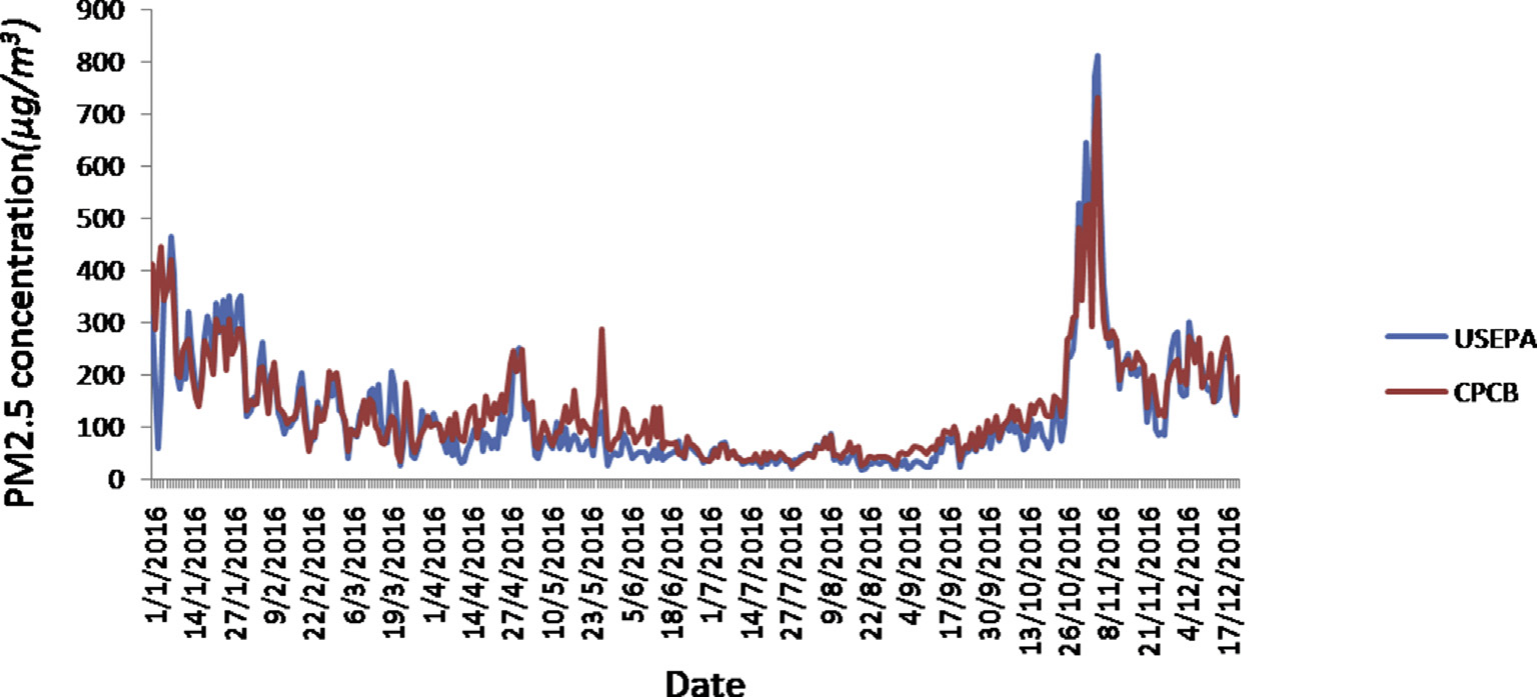
Daily variation of the US EPA and CPCB monitored PM2.5 values.

The daily concentration of fine particulate matter monitored by US EPA and CPCB from 1st January 2016 to 19th December 2016 has been taken for the present study. The data has been obtained from http://cpcb.nic.in/ and https://www.epa.gov/ for CPCB and US EPA respectively with time series in Fig. 1 and descriptive statistics shown in Table 1 [1], [3].

**Table 1:** Description of statistical parameters

	US EPA	CPCB
Mean	122.1859	135.9284
Median	84.16667	112.4575
Standard Deviation	110.502	99.11627
Kurtosis	9.121813	6.596559
Coefficient of Variation	9.04376e-1	7.2918e-1
Skewness	2.461981	2.036923

### 1.1 Study area

The U.S Embassy and Consulates manage airborne fine particulate matter monitoring. PM2.5 is a standard recognized by US EPA and permit to examine against U.S. standard measures [5]. US EPA covers Chanakyapuri area in Delhi. Central Pollution Control Board (CPCB) of India is the apex organization in country for monitoring pollution [6]. One of its monitoring stations is at RK Puram, Delhi. RK Puram and Chanakyapuri are 3.1 miles away. New Delhi, the capital of India and has Latitude, longitude coordinates as 28.644800, 77.216721 respectively. Chanakyapuri in Delhi has Latitude, longitude coordinates as 28.593853, 77.188736 and RK Puram as 28.566008 and 77.176743 respectively.

## 2 Experimental design, materials and methods

The study is divided into two sections of statistical and predictive analysis. Descriptive and inferential statistics are a vital part of data analysis. Analyzing data includes studying the statistics of data. The descriptive analysis describes big data using different measurements as indicated in Table 1.

A further step is to observe if there is any significant relation between the data considered. The correlation coefficient is a measure of the strength of the linear relationship between two such variables and is calculated as

(1) $$r_{uv}=\frac{\sum u_{i}v_{i}-m\bar{u}\bar{v}}{\left(n-1\right)\delta_{u}\delta_{v}}=\frac{m\sum u_{i}v_{i}-\sum u_{i}\sum v_{i}}{\sqrt{m\sum u_{i}^{2}-{\left(\sum u_{i}\right)}^{2}}\sqrt{m\sum v_{i}^{2}-{\left(\sum v_{i}\right)}^{2}}}$$

r_uv_ lies between −1 and +1 inclusive as discussed in Bhardwaj and Pruthi, 2016 [2]. The value of Pearson correlation is 0.933 significant at 0.01 level proving the reliability of data observed.

**Fig. 2:**
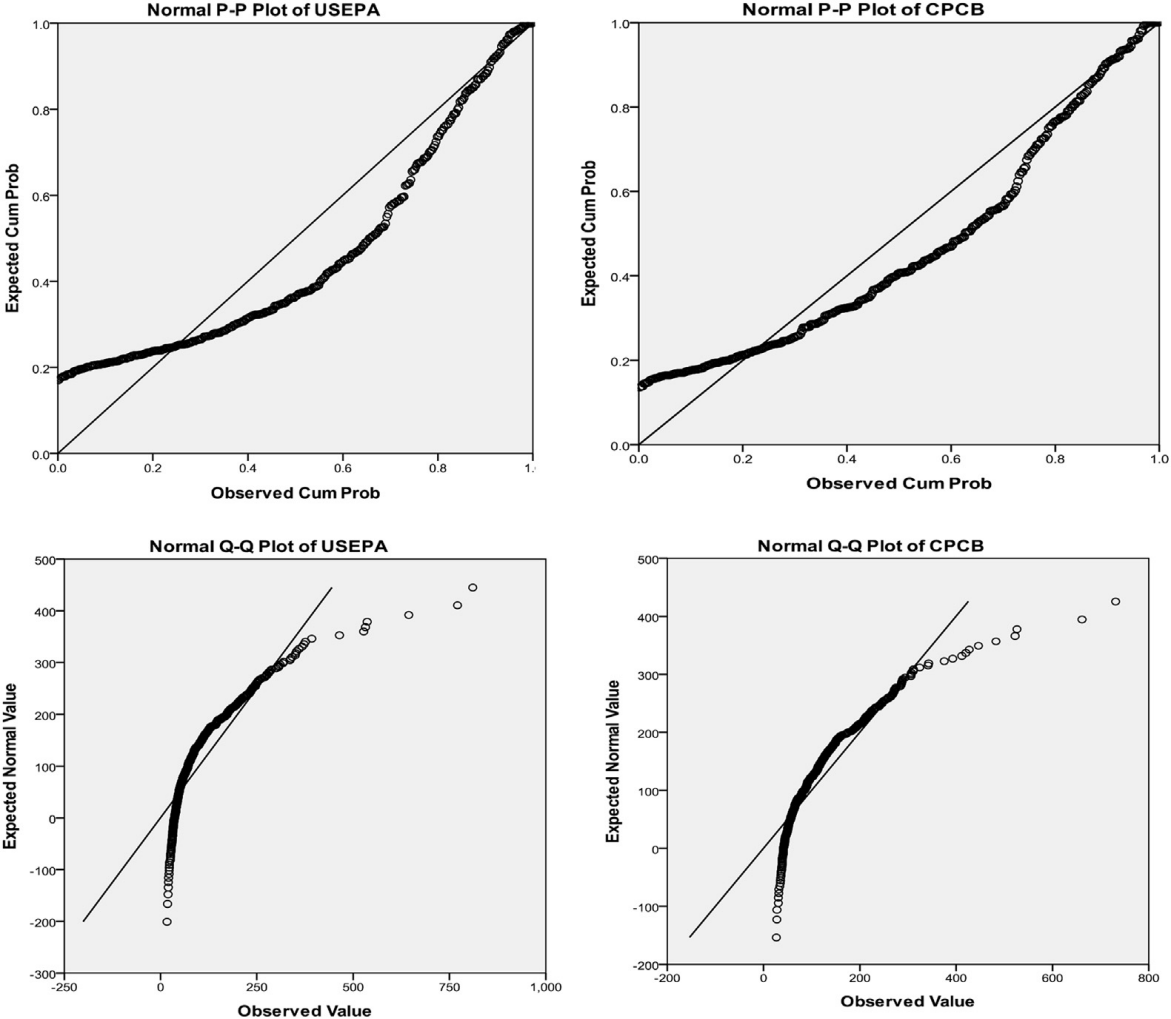
P–P plot and Q–Q plot of US EPA and CPCB PM2.5 concentrations.

Location and scale (estimated normal distribution parameters) of US EPA and CPCB data sets for unweighted cases using Blom's proportion estimation formula is calculated in Table 2. The probability-probability plot in Fig. 2 depicts deviation from the normal distribution. Location and scale values show a persistent trend for the data sets of PM2.5.

**Table 2:** Estimated Distribution Parameters of PM2.5 (US EPA and CPCB)

		US EPA	CPCB
Normal Distribution	Location	122.1859	135.9284
	Scale	110.50204	99.11627

The cases are unweighted.

The extensively used *t*-test is carried out to analyze the difference between data monitored by USEPA and CPCB. Null hypothesis, H_0_: No mean difference between USEPA and CPCB monitored data i.e. $\mu_{USEPA}-\mu_{CPCB}=0$ and alternative hypothesis, $H_{a}\text{:}\;\mu_{USEPA}-\mu_{CPCB}\neq 0\text{.}$

(2) $$t=\frac{\mu_{USEPA}-\mu_{CPCB}}{\sqrt{\frac{SD_{USEPA}^{2}+SD_{CPCB}^{2}}{N}}}$$

Calculated *t*-value is compared to *t*-value corresponding to degree of freedom (see Table 3):

(3) $$df=\frac{{\left(\frac{SD_{USEPA}^{2}+SD_{CPCB}^{2}}{N}\right)}^{2}}{\frac{1}{N-1}\left({\left(\frac{SD_{USEPA}^{2}+SD_{CPCB}^{2}}{N}\right)}^{2}-\frac{2SD_{USEPA}SD_{CPCB}}{N^{2}}\right)}$$

where, μ represents mean and *SD* standard deviation. The *F*-test statistic using one-way ANOVA is evaluated to emphasize the mean difference between two datasets (Table 4).

(4) $$F=\frac{\mathrm{regression}\;\mathrm{mean}\;\mathrm{square}\left(MSR\right)}{\mathrm{mean}\;\mathrm{square}\;\mathrm{error}\left(MSE\right)}$$

where, *MSE* is error sum of squares divided by df associated and *MSR* is regression sum of squares.

**Table 3:** *t*-test for PM2.5 (US EPA and CPCB)

Sample	N	Mean	Std. Deviation	Std. Error Mean	T	DF	p-value
USEPA	357	122.1859	110.502	5.84839	6.479	356	0.000
CPCB	357	135.9284	99.11627	5.24579

**Table 4:** One-way ANOVA for PM2.5 (US EPA and CPCB)

Source of Variation	Df	SS	MS	F	p-value
Model	1	32382.37	32382.37	2.97299	0.00
Error	710	7733453.143	10892.19		

ANOVA and *t*-test sufficiently emphasized the significant difference between PM2.5 data monitored by two major agencies US EPA and CPCB.

### 2.1 Auto regressive integrated moving average

The preferred method in time series modeling is Box-Jenkins. Box-Jenkins method constitutes three components “AR,” “I,” or “MA” thus resulting in ARIMA. An ARIMA model can be expressed as

(5) $$\left(1-\sum_{i}^{p}\theta_{i}L^{i}\right){\left(1-L\right)}^{d}y_{t}=(1+\sum_{i}^{q}\phi_{i}L^{i})\epsilon_{t}$$

The above equations were fitted to PM2.5 data. An approach of identification, estimation and diagnostic is carried out for ARIMA modeling. It defines large-scale variation in behavior of stationary time series. ARIMA is build upon present and past values of response and residuals. The main steps of ARIMA methods are: • Identification – examining the data along with calculation and drawing a graph of auto-correlation and partial auto-correlation functions. The smallest values of parameters are sought. When the value is 0, corresponding AR or MA component is not requisite in the respective model. ‘I’ component in ARIMA (trend) is examined first. The objective is to determine whether the process is stationary (d = 0) or not. If not than it has to be transformed into such. No connection between every two sequential observations implies p = 0. • Constructing models and estimating its parameters [7]. • Diagnostics and selection of model – the residuals and the quality of approximation of the model are examined. Theoretically, it is assumed that residuals are random and normally distributed. • Application of the predictive model, forecasts, analysis of dependencies, and study problem-solving capabilities [4].

Using ARIMA algorithm, PM2.5 is forecasted. Table 5, Table 6, Table 7 summarizes the output of fitted ARIMA Model.

**Table 5:** ARIMA model

	ARIMA Model	Coefficients			
		AR1	MA1	MA2	MA3
US EPA	(1,0,3)	0.982	0.142	0.236	0.22
CPCB	(1,0,2)	0.994	0.237	0.247

**Table 6:** Forecasted values

Date	US EPA				CPCB			
	Observed	Forecasted	LCL	UCL	Observed	Forecasted	LCL	UCL
20/12/2016	122.08	146.74	41.54	251.93	128.99	177.95	86.85	269.05
21/12/2016	163.34	161.8	24.43	299.17	171.13	192.67	78.39	306.96
22/12/2016	181.08	160.54	9.88	311.19	197.02	191.53	68.28	314.77

LCL and UCL stand for lower and upper confidence limits respectively.

**Table 7:** R-squared values

R-squared		PM2.5 Forecasted	
		USEPA	CPCB
PM2.5 Observed	USEPA	0.9842	0.8520
	CPCB	0.8656	0.9768

To check the accuracy of modeling following statistics are used:

(6) $$R^{2}=1-\frac{\text{sum}\;\text{of}\;\text{squared}\;\text{error}\;\text{of}\;\text{model}}{\text{sum}\;\text{of}\;\text{squared}\;\text{error}\;\text{of}\;\text{baseline}\;\text{model}}$$

### 2.2 Air quality index

**Fig. 3:**
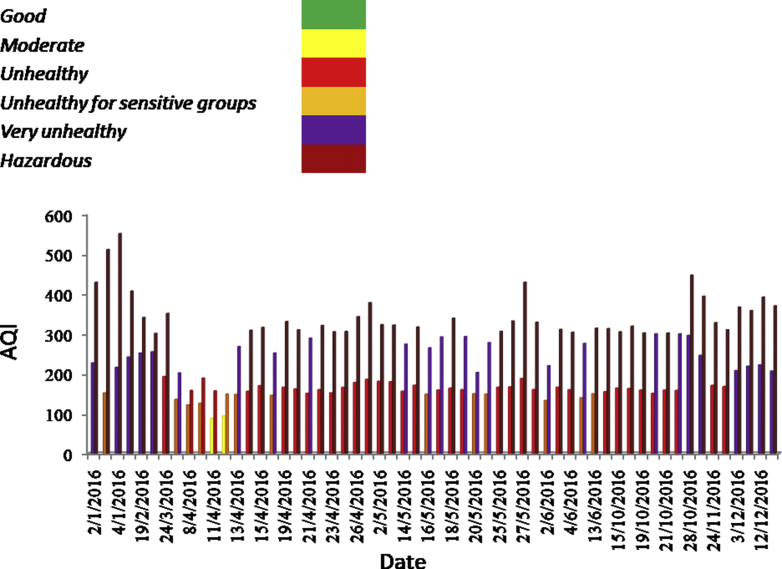
Air Quality Index corresponding to PM2.5 concentration monitored by US EPA and CPCB.

Air Quality Index is not just a number signifying the quality of air but also explaining what we are inhaling. AQI was introduced in 1968. The objective was to aware public about deteriorating air quality and raise alarm in order to take precautionary measures. AQI is calculated using www.cpcb.nic.in. and www.epa.gov. To focus on the effects of monitoring variability air quality index is calculated. AQI is divided into the following categories:

In Fig. 3 AQI is represented for those days in which they fall in different categories. The first and second bar in Fig. 3 represents calculated AQI corresponding to USEPA and CPCB data respectively. In a short span of 354 days AQI falls in different categories for 58 days.

### 2.3 Concluding remark

It is noted that PM2.5 monitored by US EPA and CPCB show a significant difference. Mathematically, US EPA measured PM2.5 data can be formed from CPCB by adding 13.7425 ± 7.856331729 and vice-versa by subtracting. AQI calculated fall in different categories as per National Standards. This might have lead to the issuance of wrong public health advisories in the past and if this difference is not observed as carried out in the present study, it may have a significant adverse impact on human health in future as well.
